# Supplementary material for: Vitamin D Modified DSS-Induced Colitis in Mice via STING Signaling Pathway
Source: Biology (Basel). 2025 Jun 18;14(6):715. doi: 10.3390/biology14060715 (PMC12190092; doi:10.3390/biology14060715)
Supplement: Supplementary file 1 [file biology-14-00715-s001.zip › Supplementary Materials S1: 16s-report of gut microbiota/Groups/diff_analysis/UnivarTestFamily/figure/barplot/top10.pdf]

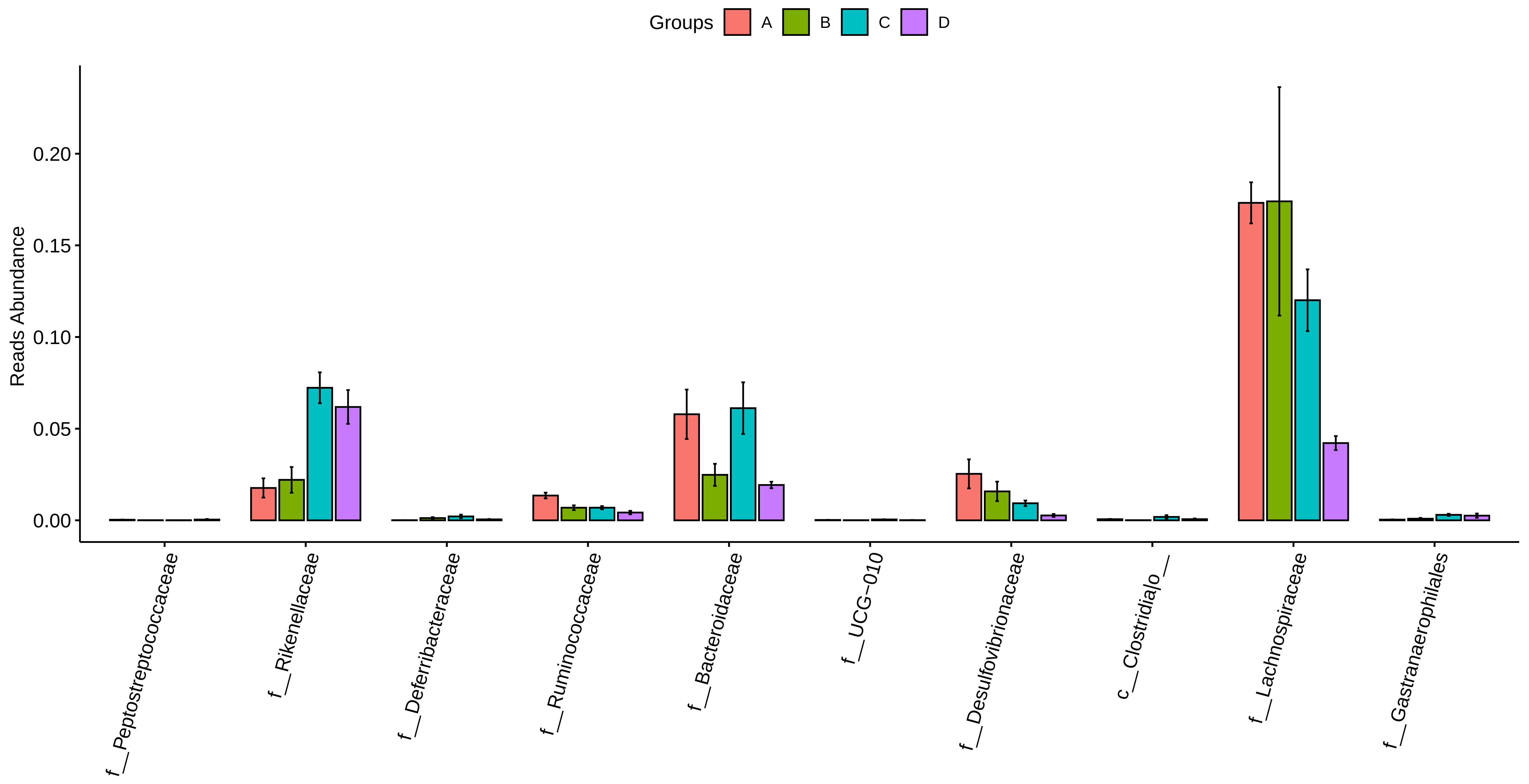
Groups
A
B
C
D
Reads Abundance
0.20
0.15
0.10
0.05
0.00
f__Peptostreptococcaceae
f__Rikenellaceae
f__Deferribacteraceae
f__Ruminococcaceae
f__Bacteroidaceae
f__UCG−010
f__Desulfovibrionaceae
c__Clostridia|o__
f__Lachnospiraceae
f__Gastranaerophilales
